# Association between malnutrition risk and hemorrhagic transformation in patients with acute ischemic stroke

**DOI:** 10.3389/fnut.2022.993407

**Published:** 2022-10-05

**Authors:** Cheng-Xiang Yuan, Yi-Ni Zhang, Xuan-Yu Chen, Bei-Lei Hu

**Affiliations:** ^1^Department of Neurology, The Second Affiliated Hospital and Yuying Children's Hospital of Wenzhou Medical University, Wenzhou, China; ^2^Department of Gastroenterology, The First Affiliated Hospital of Wenzhou Medical University, Wenzhou, China

**Keywords:** malnutrition risk, acute ischemic stroke, hemorrhagic transformation, controlling nutritional status (CONUT) score, prognosis, risk factors

## Abstract

**Background and aims:**

Malnutrition is a prevalent problem occurring in different diseases. Hemorrhagic transformation (HT) is a severe complication of acute ischemic stroke (AIS). Few studies have evaluated the association between malnutrition risk and hemorrhagic transformation in patients with acute stroke. We aim to investigate the influence of malnutrition risk on the risk of hemorrhagic transformation in patients with AIS.

**Methods:**

A total of 182 consecutive adults with HT and 182 age- and sex-matched patients with stroke were enrolled in this study. The controlling nutritional status (CONUT) score was calculated to evaluate the malnutrition risk. HT was detected by follow-up imaging assessment and was radiologically classified as hemorrhagic infarction type 1 or 2 or parenchymal hematoma type 1 or 2. Logistic regression models were conducted when participants were divided into different malnutrition risk groups according to the objective nutritional score to assess the risk for HT.

**Results:**

The prevalence of moderate to severe malnutrition risk in patients with AIS was 12.5%, according to the CONUT score. Univariate analysis showed that the CONUT score is significantly higher in patients with HT than those without HT. After adjusting for potential covariables, the patients with mild risk and moderate to severe malnutrition risk were associated with a higher risk of HT compared to the patients in the normal nutritional status group [odds ratio, 3.180 (95% CI, 1.139–8.874), *P* = 0.027; odds ratio, 3.960 (95% CI, 1.015–15.453), *P* = 0.048, respectively].

**Conclusion:**

Malnutrition risk, measured by CONUT score, was significantly associated with an increased risk of hemorrhagic transformation in patients with AIS.

## Introduction

Malnutrition has emerged as a common condition in older patients with chronic diseases that can lead to poor prognoses (1–3). It is connected with increased complications, a longer hospital stay, higher cost of hospitalization, and mortality (4–6). However, malnutrition is often overlooked and untreated in older adults.

The reported prevalence of malnutrition at admission after stroke has varied from 9 to 20% (7, 8). Due to various stroke-related and age-related complications, it is challenging and essential to evaluate the nutritional status of patients with stroke in routine clinical practice. Nevertheless, it could be difficult to assess the nutritional condition using standard malnutrition screening tools because of the requirements for professional assistance and the complexity of measurement procedures (9). Previous studies have advocated the use of simple risk scoring strategies for rapid assessment of patients with acute ischemic stroke (AIS) at risk of malnutrition, which encompassed both manifest and potential malnutrition (8, 10). Recently, several objective malnutrition scores, including the controlling nutritional status (CONUT) (11) score and the Geriatric Nutritional Risk Index (GNRI) (12), have been widely used to evaluate the risk of malnutrition in different diseases (13, 14). The CONUT score and GNRI share the same serum albumin component, whereas the CONUT score incorporates more serum nutritional indices, including albumin, lymphocyte count, and cholesterol levels. These blood compositions play a role in the clinical outcomes of patients with AIS. Serum albumin, for instance, can reduce hematocrit levels and influence erythrocyte aggregation, thereby increasing cerebral blood flow by blood dilution, which could rescue more neurons in the ischemic penumbra area and reduce injury (15). Besides, increased vascular permeability is a crucial cause of the lowered concentration of albumin commonly found in acute and chronic diseases (16). A previous study has demonstrated that human albumin contributes to the maintenance of vascular integrity and normal capillary permeability (17).

Hemorrhagic transformation (HT), the bleeding of the ischemic brain area after stroke, is a severe and notable complication occurring in patients with AIS (18, 19). It can potentially contribute to the clinical deterioration of AIS patients with increased stroke disability rates and mortality, further imposing the economic and social burden on these people (20–22). Thus, an improved understanding and active exploration of the risk factors for HT would be of great clinical value in identifying those at increased risk of HT, allowing clinicians to take preventive or therapeutic measures and restore the blood flow of the ischemic brain without the occurrence of this complication, and consequently enhance the quality of life for patients with AIS. Until now, several risk factors for HT have been identified in previous studies, including age (23), atrial fibrillation (24), elevated blood pressure (25), thrombolytic treatment (26), and symptom severity (27). In fact, research has shown that malnutrition was associated with a negative embolic-hemorrhagic balance in anticoagulated patients and a clinical predictor of poor prognosis in octogenarian patients with atrial fibrillation (4). A recent study of 8,698 patients with AIS suggested that malnutrition risk was associated with a higher risk of major disability and long-term death (10). However, data regarding the effect of malnutrition risk on the risk of HT are lacking. Hence, this study aimed to investigate the association between malnutrition risk and HT in patients with AIS and hopefully aid physicians in clinical prediction.

## Materials and methods

### Study population

We enrolled consecutive participants from the Second Affiliated Hospital and Yuying Children's Hospital of Wenzhou Medical University clinical database of HT between January 2009 and April 2022 in this retrospective study. This study was approved by the Institutional Review Board and Research Ethics Committee of the Second Affiliated Hospital and Yuying Children's Hospital of Wenzhou Medical University. No requirement for informed consent because this study was retrospective, and all included data were anonymous. The human data collection and the article writing were in accordance with the ethical guidelines of the Declaration of Helsinki.

Patients were included if they (a) were aged between 18 and 90 years, (b) were admitted within 7 days of the onset of symptoms, and (c) were diagnosed as AIS by a brain computed tomography (CT) or magnetic resonance imaging (MRI) scan during hospitalization or right before it. Patients were excluded if they (a) were identified as having a transient ischemic attack (TIA) or hemorrhagic stroke, (b) were unable to check for repeatable CT or MRI, or (c) lacked laboratory data.

### Data collection

Demographic characteristics, vascular risk factors, and radiological images were obtained by trained research coordinators. The baseline vascular risk factors included hypertension, diabetes mellitus, dyslipidemia, atrial fibrillation, previous history of stroke, smoking status, and current alcohol consumption. Laboratory data in the blood samples (including white cell count, lymphocyte count, fibrinogen, serum albumin, creatinine, and total cholesterol) and blood pressure measurements were collected within 24 h after admission. They were measured at the clinical laboratory in our hospital using routine laboratory methods. All the laboratory measurements were conducted by experienced laboratory technicians who were blinded to the baseline characteristics and clinical outcomes of the study participants. Treatment information on all the patients with AIS, including thrombolytic therapy, anticoagulant, and antiplatelet therapies before admission, was gathered. The size of the infarction area involving more than one-half of the middle cerebral artery territory was defined as large (28, 29). Stroke etiology was classified according to the TOAST (Trial of ORG 10172 in Acute Stroke Treatment) criteria (30). The stroke severity was evaluated by well-trained neurologists using the National Institute of Health Stroke Scale (NIHSS) (31) score within 24 h of admission.

### Nutritional screening score

Patients were screened for malnutrition risk using the CONUT score (Table 1). The CONUT score was calculated from three variables to assess the malnutrition risk, including the level of lymphocyte count, serum albumin, and total cholesterol. The components of CONUT can be categorized into four groups, respectively, according to the levels in the blood and given corresponding scores. A total score of 0–1 was considered normal; scores of 2–4, 5–8, and 9–12 were classified as mild, moderate, and severe malnutrition risk, respectively.

**Table 1:** Details of the CONUT scoring system.

**Parameter**	**Normal**	**Mild**	**Moderate**	**Severe**
Albumin, g/dl	≥3.5	3.0–3.4	2.5–2.9	<2.5
Score	Zero	Two	Four	Six
Lymphocyte count, 10^9^/l	≥1.60	1.20–1.59	0.80–1.19	<0.8
Score	Zero	One	Two	Three
Total cholesterol, mg/dl	≥180	140–179	100–139	<100
Score	Zero	One	Two	Three
CONUT, points	Zero to one	Two to four	Five to eight	Nine to twelve

CONUT, controlling nutritional status score.

In this study, we combined moderate and severe malnutrition status as a moderate-severe malnutrition risk for analysis.

### Definition of “HT”

Patients were diagnosed with HT by a follow-up CT or MRI performed within 24 h and 7 days (±2) from the onset of stroke. In addition, image examinations were conducted whenever the patients' clinical condition appeared to worsen for quick detection of the presence of HT. Two experienced neurologists, who were blinded to the clinical characteristics of each patient, retrospectively confirmed the results of the official neuroradiology report to agree on the presence and subtypes of HT. Hemorrhagic events were further radiologically divided into hemorrhagic infarction (HI) (types 1 and 2) and parenchymal hematoma (PH) (types 1 and 2) according to the recommendations of the European Cooperative Acute Stroke Study (32).

### Statistical analyses

For descriptive analysis, continuous parameters were presented as means with standard deviation or median with interquartile range, while categorical variables were reported as frequencies (percentages). Statistic comparisons were performed with different methods on the basis of the nature of the data. The chi-square test, or Fisher's exact test, was used for categorical variables. The normally distributed variables were compared using the student's *t*-test and analysis of variance (ANOVA), while the non-normally distributed variables were compared using the Mann–Whitney *U*-test and the Kruskal–Wallis *H*-test. The CONUT score was used to evaluate the prevalence of malnutrition risk at admission. Identified variables related to HT in the univariable analysis and main baseline confounders were selectively entered into the multivariable analyses with a conditional logistic regression model to explore significant predictors of HT. According to the malnutrition risk assessed by the CONUT score, the normal group was selected as the reference to compare the risk for HT, HI, and PH between the three malnutrition risk groups. All statistical analyses were conducted with SPSS for Windows, version 22.0 (SPSS Inc., Chicago, IL, USA) and GraphPad Prism, version 8.0.2. Additionally, a two-sided *P*-value < 0.05 was judged to be statistically significant.

## Results

### Baseline characteristics

At baseline, a total of 182 consecutive participants with HT and 182 age- and sex-matched ischemic stroke patients without HT were enrolled in this study, among whom 345 (94.8%) patients had information on all the lymphocyte count, total cholesterol, and albumin data. The median age of these included patients was 74.0 (interquartile range, 66.0–81.0) years, ranging from 28 to 90. Most of these patients were men (56.3%). The median National Institute of Health Stroke Scale score (NIHSS) was 6.0 (interquartile range, 2.0–14.0). Among the patients diagnosed with hemorrhagic transformation, hemorrhagic infarct type 1 (HI-1) occurred in 30 (16.5%) patients, hemorrhagic infarct type 2 (HI-2) in 80 (44.0%), parenchymal hematoma type 1 (PH-1) in 49 (26.9%), and parenchymal hematoma type 2 (PH-2) in 23 (12.6%). The subcategorization was based on imaging features.

Table 2 shows the baseline characteristics and clinical features of included participants with and without HT. In this study sample, patients with higher baseline white cell counts, fibrinogen, or higher initial NIHSS scores were more likely to undergo HT, while those with elevated lymphocyte counts or albumin were less likely to undergo HT. In comparison to patients without HT, patients in the HT group were likely to identify a larger infarction area, a higher proportion of history of atrial fibrillation, and anticoagulant therapies before admission.

**Table 2:** Baseline characteristics of the AIS patients with and without HT.

**Variables**	**Non-HT (*n* = 182)**	**HT (*n* = 182)**	***P*-value**
**Patient characteristics**			
Age (years)	74.0 (66.0–80.0)	75.0 (66.0–81.0)	0.713
Male, *n* (%)	101 (55.5%)	104 (57.1%)	0.751
BMI (kg/m^2^)	24.0 (22.2–26.9)	22.4 (20.8–25.8)	0.009
History of stroke, *n* (%)	45 (24.7%)	42 (23.1%)	0.712
History of atrial fibrillation, *n* (%)	17 (9.3%)	55 (30.2%)	<0.001
History of hypertension, *n* (%)	140 (76.9%)	129 (70.9%)	0.189
History of diabetes, *n* (%)	54 (29.7%)	53 (29.1%)	0.908
History of dyslipidemia, *n* (%)	4 (2.2%)	7 (3.8%)	0.358
Smoking status, *n* (%)			0.626
Nonsmokers	132 (72.5%)	140 (76.9%)	
Former smokers	17 (9.3%)	14 (7.7%)	
Current smokers	33 (18.1%)	28 (15.4%)	
Current drinking, *n* (%)	26 (14.3%)	30 (16.5%)	0.561
**Biochemistry and vital signs on admission**			
Baseline SBP (mmHg)	154.5 ± 21.2	152.9 ± 23.1	0.530
White cell count, 10^9^/L	7.0 (5.8–8.4)	9.2 (7.1–11.1)	<0.001
Lymphocyte count, 10^9^/L	1.6 (1.2–2.0)	1.3 (0.9–1.7)	<0.001
Albumin, (g/L)	40.0 (37.4–41.9)	37.5 (34.5–40.4)	<0.001
Fibrinogen (g/L)	3.4 (2.9–3.8)	3.6 (2.9–4.3)	0.017
Creatinine (μmol/L)	64.9 (54.6–76.7)	66.7(57.2–83.0)	0.118
Total cholesterol (mmol/L)	4.4 (3.7–5.0)	4.2 (3.4–5.0)	0.269
Large size of the infarction area, *n* (%)	19 (10.4%)	91 (50.0%)	<0.001
NIHSS on admission, median (IQR)	4.0 (2.0–8.0)	11.0 (4.0–18.0)	<0.001
Thrombolytic therapy, *n* (%)	26 (14.3%)	39 (21.4%)	0.075
Anticoagulants therapy before admission, *n* (%)	4 (2.2%)	15 (8.2%)	0.010
Antiplatelets therapy before admission, *n* (%)	12 (6.6%)	10 (5.5%)	0.660
CONUT, median (IQR)	2.0 (1.0–3.0)	3.0 (1.0–4.0)	**<0.001**
**Malnutrition**			**<0.001**
Normal, *n* (%)	88 (49.4%)	47 (28.1%)	
Mild, *n* (%)	78 (43.8%)	89 (53.3%)	
Moderate, *n* (%)	12 (6.7%)	28 (16.8%)	
Severe, *n* (%)	0 (0.0%)	3 (1.8%)	
**Stroke mechanisms**			<0.001
Atherosclerotic, *n* (%)	134 (73.6%)	75 (41.2%)	
Cardioembolic, *n* (%)	27 (14.8%)	101 (55.5%)	
Lacunar, *n* (%)	21 (11.5%)	0 (0.0%)	
Other causes, *n* (%)	0 (0.0%)	6 (3.3%)	

AIS, acute ischemic stroke; HT, hemorrhagic transformation; BMI, body mass index; SBP, systolic blood pressure; NIHSS, National Institutes of Health Stroke Scale; CONUT, Controlling Nutritional Status Score; and IQR, interquartile range. The bold values indicate the P values to highlight the analysis results.

### Malnutrition risk according to CONUT score

The median CONUT score was 2.0 (interquartile range, 1.0–3.0; minimum, 0; maximum, 11) in the total population. By CONUT score, 167 (48.4%) patients had mild malnutrition, 40 (11.6%) patients had moderate malnutrition, and three (0.9%) patients had severe malnutrition. The baseline demography and disease characteristics of the patients with different malnutrition risks based on CONUT scores are displayed in Table 3. Participants in the normal group were generally younger (72.0 years, interquartile range: 65.0–78.0). Compared to those with the presence of other malnutrition risk groups, patients without malnutrition had higher lymphocyte counts, total cholesterol, and albumin and were less likely to undergo anticoagulant therapies before admission. Meanwhile, a lower proportion of the large size of infarction, previous stroke, and atrial fibrillation, as well as lower initial NIHSS, were found in the normal group. Baseline characteristics of the patients according to the subcategorized groups of HT in the total population are shown in Table 4.

**Table 3:** Baseline characteristics of the study participants stratified by malnutrition.

**Variables**	**Normal**	**Mild risk**	**Moderate-severe risk**	***P***	***P*-value**
	**(*n* = 135)**	**(*n* = 167)**	**(*n* = 43)**	**for trend**	
**Patient characteristics**				
Age (years)	72.0 (65.0–78.0)	76.0 (67.0–81.0)	78.0 (73.0–84.0)	<0.001	<0.001
Male, *n* (%)	73 (54.1%)	97 (58.1%)	22 (51.2%)	0.974	0.642
BMI (kg/m^2^)	24.5 (22.1–27.1)	23.5 (21.7–25.4)	21.2 (19.9–23.8)	0.546	0.024
History of stroke, *n* (%)	21 (15.6%)	49 (29.3%)	14 (32.6%)	0.004	0.009
History of atrial fibrillation, *n* (%)	16 (11.9%)	36 (21.6%)	15 (34.9%)	0.001	0.002
History of hypertension, *n* (%)	96 (71.1%)	127 (76.0%)	31 (72.1%)	0.617	0.608
History of diabetes, *n* (%)	46 (34.1%)	47 (28.1%)	7 (16.3%)	0.028	0.077
History of dyslipidemia, *n* (%)	2 (1.5%)	7 (4.2%)	2 (4.7%)	0.178	0.348
Smoking status, *n* (%)				0.194	0.372
Nonsmokers	98 (72.6%)	126 (75.4%)	35 (81.4%)		
Former smokers	13 (9.6%)	12 (7.2%)	5 (11.6%)		
Current smokers	24 (17.8%)	29 (17.4%)	3 (7.0%)		
Current drinking, *n* (%)	24 (17.8%)	24 (14.4%)	4 (9.3%)	0.167	0.376
**Biochemistry and vital signs on admission**				
Baseline SBP (mmHg)	155.0 ± 23.4	152.9 ± 20.9	151.1 ± 22.2	0.256	0.361
White cell count, 10^9^/L	7.5 (6.2–9.7)	7.9 (6.1–10.3)	7.7 (5.9–11.6)	0.580	0.410
Lymphocyte count, 10^9^/L	1.9 (1.6–2.2)	1.2 (1.0–1.6)	0.7 (0.6–1.0)	<0.001	<0.001
Albumin, (g/L)	40.3 (38.3–42.1)	38.3 (36.0–40.6)	33.4 (30.9–34.7)	0.094	<0.001
Fibrinogen (g/L)	3.5 (3.0–4.0)	3.4 (2.9 - 4.2)	3.4 (2.7–4.0)	0.299	0.719
Creatinine (μmol/L)	63.4 (53.7–74.0)	64.7 (56.1–82.8)	69.2 (58.7–86.0)	0.021	0.091
Total cholesterol (mmol/L)	4.8 (4.3–5.4)	4.0 (3.3–4.7)	3.4 (2.8–4.2)	<0.001	<0.001
Large size of the infarction area, *n* (%)	25 (18.5%)	55 (32.9%)	17 (39.5%)	0.001	0.004
NIHSS on admission, median (IQR)	3.0 (2.0–9.0)	7.0 (3.0–15.0)	12.0 (5.0–21.0)	<0.001	<0.001
Thrombolytic therapy, *n* (%)	23 (17.0%)	31 (18.6%)	4 (9.3%)	0.446	0.349
Anticoagulants therapy before admission, *n* (%)	3 (2.2%)	8 (4.8%)	7 (16.3%)	0.001	0.005
Antiplatelets therapy before admission, *n* (%)	7 (5.2%)	11 (6.6%)	3 (7.0%)	0.590	0.815
CONUT, median (IQR)	1.0 (0.0–1.0)	3.0 (2.0–3.0)	6.0 (5.0–7.0)	<0.001	<0.001
HT, *n* (%)	47 (34.8%)	89 (53.3%)	31 (72.1%)	<0.001	**<0.001**
**Stroke mechanisms**				0.041	<0.001
Atherosclerotic, *n* (%)	91 (67.4%)	97 (58.1%)	15 (34.9%)		
Cardioembolic, *n* (%)	30 (22.2%)	60 (35.9%)	26 (60.5%)		
Lacunar, *n* (%)	13 (9.6%)	6 (3.6%)	2 (4.7%)		
Other causes, *n* (%)	1 (0.7%)	4 (2.4%)	0 (0.0%)		

AIS, acute ischemic stroke; HT, hemorrhagic transformation; BMI, body mass index; SBP, systolic blood pressure; NIHSS, National Institutes of Health Stroke Scale; CONUT, Controlling Nutritional Status Score; and IQR, interquartile range. The bold values indicate the P values to highlight the analysis results.

**Table 4:** Baseline characteristics of the patients according to the subcategorized groups of HT.

**Variables**	**non-HT (*n* = 182)**	**HI (*n* = 110)**	**PH (*n* = 72)**	***P*-value**
**Patient characteristics**				
Age (years)	74.0 (66.0–80.0)	73.0 (64.0–80.0)	77.0 (67.0–81.0)	0.195
Male, *n* (%)	101 (55.5%)	62 (56.4%)	42 (58.3%)	0.919
BMI (kg/m^2^)	24.0 (22.2–26.9)	22.4 (21.2–26.0)	22.4 (20.1–26.3)	0.031
History of stroke, *n* (%)	45 (24.7%)	25 (22.7%)	17 (23.6%)	0.926
History of atrial fibrillation, *n* (%)	17 (9.3%)	26 (23.6%)	29 (40.3%)	<0.001
History of hypertension, *n* (%)	140 (76.9%)	75 (68.2%)	54 (75.0%)	0.250
History of diabetes, *n* (%)	54 (29.7%)	34 (30.9%)	19 (26.4%)	0.802
History of dyslipidemia, *n* (%)	4 (2.2%)	6 (5.5%)	1 (1.4%)	0.260
Smoking status, *n* (%)				0.607
Nonsmokers	132 (72.5%)	81 (73.6%)	59 (81.9%)	
Former smokers	17 (9.3%)	9 (8.2%)	5 (6.9%)	
Current smokers	33 (18.1%)	20 (18.2%)	8 (11.1%)	
Current drinking, *n* (%)	26 (14.3%)	19 (17.3%)	11 (15.3%)	0.790
**Biochemistry and vital signs on admission**				
Baseline SBP (mmHg)	154.5 ± 21.2	152.5 ± 23.5	153.5 ± 22.5	0.803
White cell count, 10^9^/L	7.0 (5.8–8.4)	8.8 (6.8–11.0)	9.3 (7.2–12.4)	<0.001
Lymphocyte count, 10^9^/L	1.6 (1.2–2.0)	1.3 (1.0–1.9)	1.2 (0.8–1.6)	<0.001
Albumin, (g/L)	40.0 (37.4–41.9)	37.7 (35.3–40.5)	37.3 (33.9–40.4)	<0.001
Fibrinogen (g/L)	3.4 (2.9–3.8)	3.5 (2.9–4.3)	3.7 (2.9–4.4)	0.053
Total cholesterol (mmol/L)	4.4 (3.7–5.0)	4.2 (3.4–5.0)	4.3 (3.6–4.9)	0.499
Large size of the infarction area, *n* (%)	19 (10.4%)	52 (47.3%)	39 (54.2%)	<0.001
NIHSS on admission, median (IQR)	4.0 (2.0–8.0)	8.0 (3.0–14.0)	15.0 (10.0–24.0)	<0.001
Thrombolytic therapy, *n* (%)	26 (14.3%)	25 (22.7%)	14 (19.4%)	0.175
Anticoagulants therapy before admission, *n* (%)	4 (2.2%)	9 (8.2%)	6 (8.3%)	0.021
Antiplatelets therapy before admission, *n* (%)	12 (6.6%)	5 (4.5%)	5 (6.9%)	0.739
CONUT, median (IQR)	2.0 (1.0–3.0)	2.0 (1.0–4.0)	3.0 (2.0–4.0)	**<0.001**
**Malnutrition**				**<0.001**
Normal, *n* (%)	88 (49.4%)	32 (30.8%)	15 (23.8%)	
Mild, *n* (%)	78 (43.8%)	56 (53.8%)	33 (52.4%)	
Moderate-severe, *n* (%)	12 (6.7%)	16 (15.4%)	15 (23.8%)	
**Stroke mechanisms**				<0.001
Atherosclerotic, *n* (%)	134 (73.6%)	50 (45.5%)	25 (34.7%)	
Cardioembolic, *n* (%)	27 (14.8%)	55 (50.0%)	46 (63.9%)	
Lacunar, *n* (%)	21 (11.5%)	0 (0.0%)	0 (0.0%)	
Other causes, *n* (%)	0 (0.0%)	5 (4.5%)	1 (1.4%)	

AIS, acute ischemic stroke; HT, hemorrhagic transformation; HI, hemorrhagic infarct; PH, parenchymal hematoma; BMI, body mass index; SBP, systolic blood pressure; NIHSS, National Institutes of Health Stroke Scale; and CONUT, controlling nutritional status score. The bold values indicate the P values to highlight the analysis results.

### Association of malnutrition and hemorrhagic transformation

**Figure 1:**
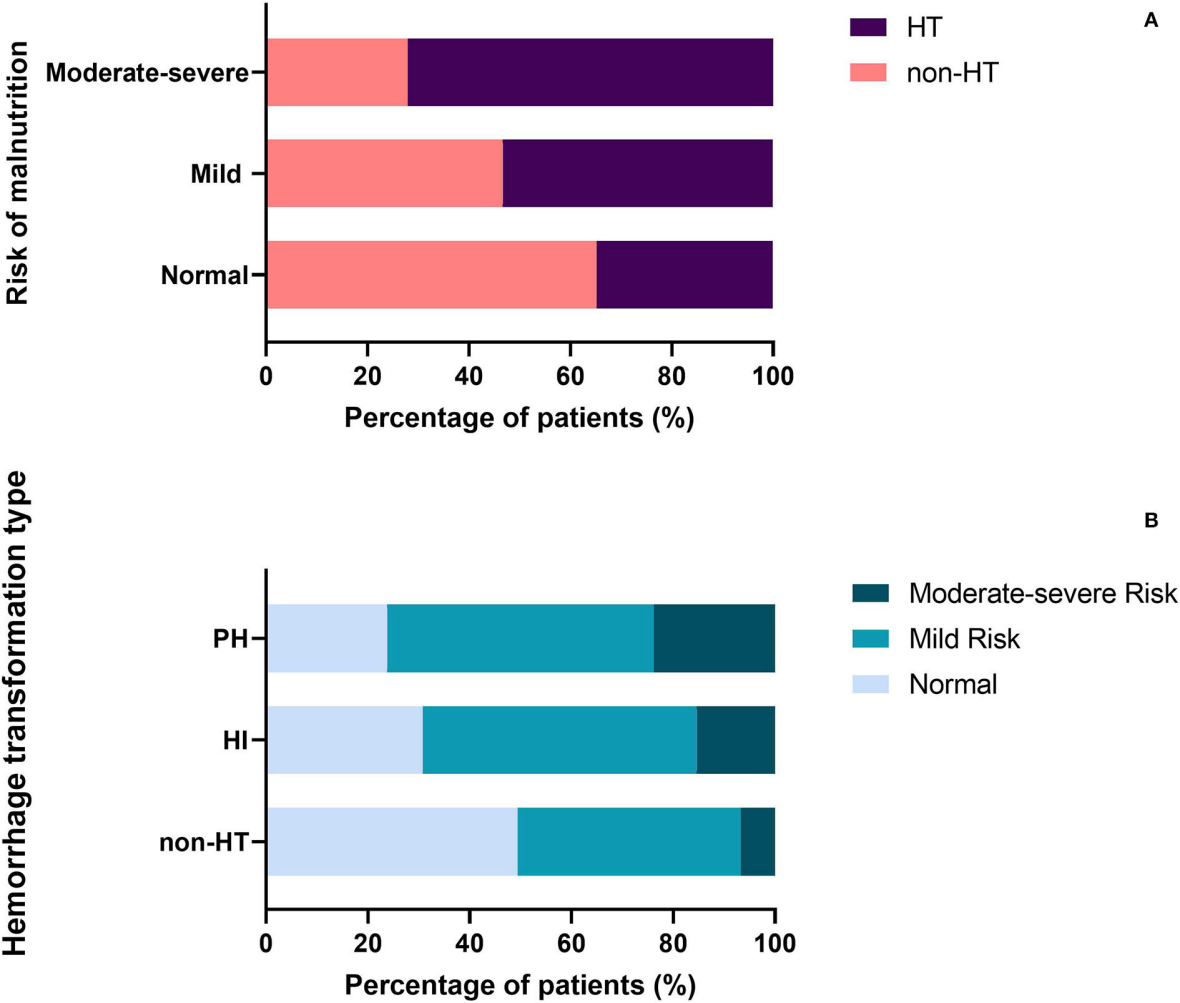
**(A)** Percentage of patients with HT in the different malnutrition risk groups. **(B)** Percentage of patients with different malnutrition risks across three subcategorized groups of HT. HT, hemorrhagic transformation; HI, hemorrhagic infarct; and PH, parenchymal hematoma.

**Figure 2:**
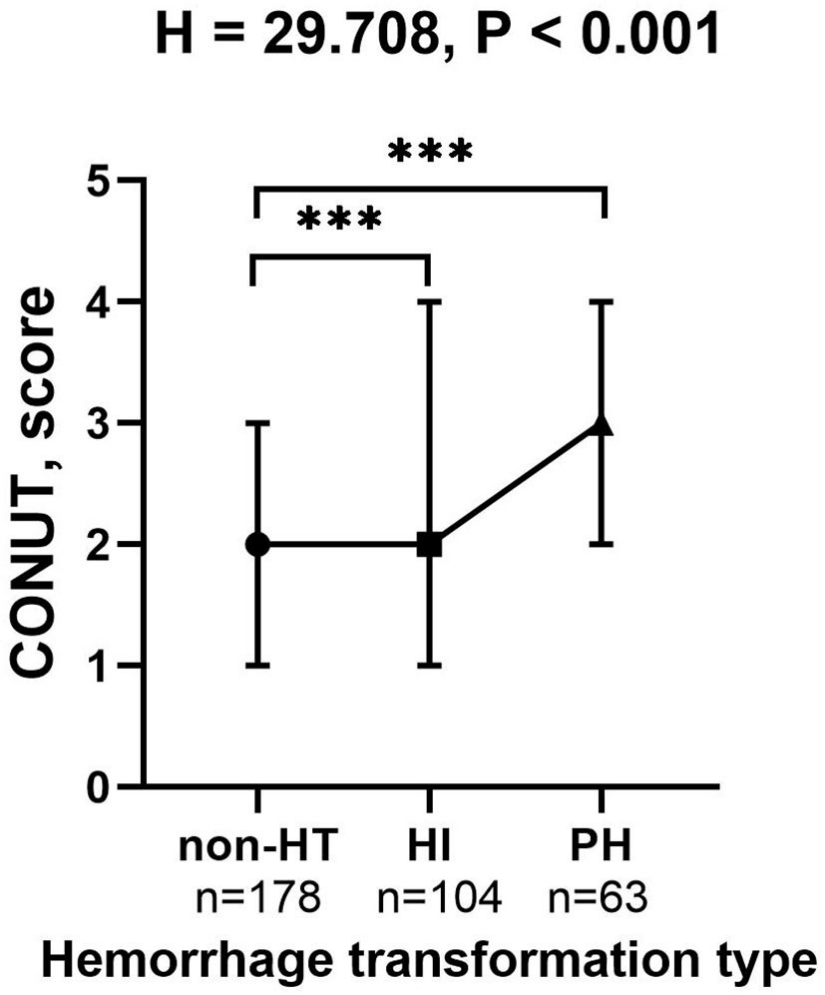
The CONUT score in the subcategorized groups of HT. Each data point and error bar represent the median and interquartile range of the CONUT score of the subcategorized groups of HT. CONUT, controlling nutritional status score; HT, hemorrhagic transformation; HI, hemorrhagic infarct; and PH, parenchymal hematoma. ***P < 0.001.

The proportion of patients diagnosed with HT was significantly different among three malnutrition risk groups using the chi-square test (χ^2^ = 21.243, *P* < 0.001; Table 3). Compared to the normal group, the proportion of HT patients was significantly higher in the mild risk group and moderate-severe risk group after the Bonferroni modification (53.3 vs. 34.8%, chi-square test *P* = 0.001; 72.1 vs. 34.8%, chi-square test *P* < 0.001, respectively, Figure 1A). However, there is no significant difference in the proportion of patients with HT between patients in the mild and moderate-severe malnutrition risk groups (53.3 vs. 72.1%, chi-square test *P* = 0.026). The prevalence of different malnutrition risks across three subcategorized groups of HT was significantly different (χ^2^ = 23.980, chi-square test *P* < 0.001; Figure 1B). Patients in the HT group had significantly higher CONUT scores than those without HT [3.0 (1.0–4.0) vs. 2.0 (1.0–3.0), *P* < 0.001; Table 2]. Additionally, CONUT scores were significantly different among three subcategorized groups of HT using the Kruskal–Wallis Test (H = 29.708, *P* < 0.001; Table 4). After the Bonferroni modification, the CONUT score was significantly higher in the HI group and PH group compared to that in the non-HT group [2.0 (1.0–4.0) vs. 2.0 (1.0–3.0), *P* < 0.001; 3.0 (2.0–4.0) vs 2.0 (1.0–3.0), *P* < 0.001, respectively, Figure 2]. However, there is no significant difference in the CONUT score between patients with HI and PH [2.0 (1.0–4.0) vs. 3.0 (2.0–4.0), *P* = 0.329].

**Figure 3:**
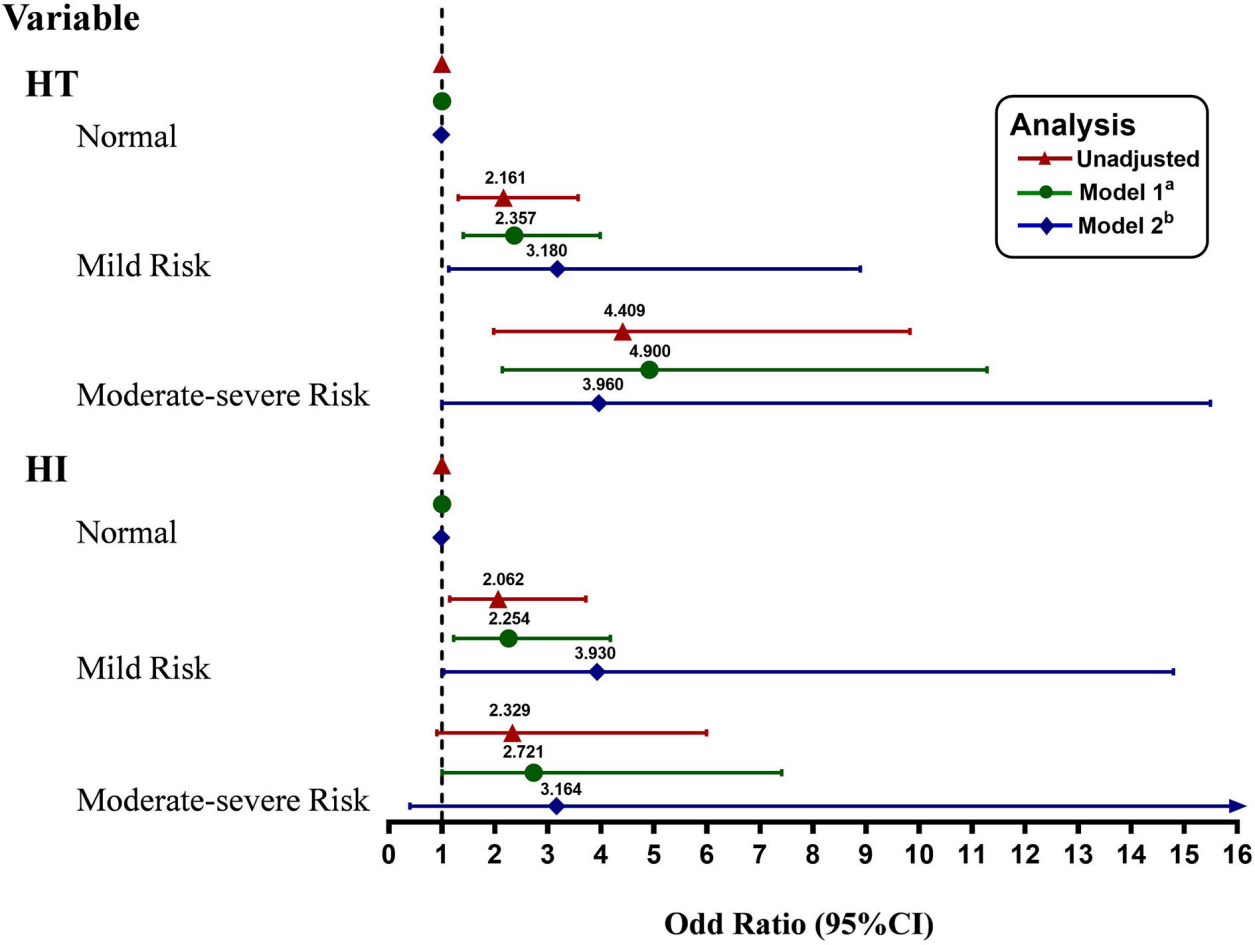
Multivariate adjusted odds ratios for the association between the different malnutrition risks and the subcategorized groups of HT (including HT and HI, respectively). Reference OR (1.000) is the normal nutritional status group. ^a^Model 1: adjusted for age and sex. ^b^Model 2 was additionally adjusted for hypertension, atrial fibrillation, diabetes, dyslipidemia, history of stroke, smoking status, current alcohol drinking, size of the infarction area, stroke mechanism, SBP, baseline white cell count, fibrinogen, creatinine, baseline NIHSS score, thrombolytic therapy, and anticoagulant and antiplatelet use before admission. CI, confidence level; HT, hemorrhagic transformation; and HI, hemorrhagic infarct.

According to the CONUT scores calculated by the equation as the categorical variable grouped previously mentioned, we performed the conditional logistic regression models. Similarly, univariable analyses indicated that mild malnutrition risk and moderate to severe malnutrition risk showed significant associations with an increased risk of HT without adjustment [OR = 2.161, (95% CI = 1.309–3.567), *P* = 0.003; OR = 4.409, (95% CI = 1.978–9.829), *P* < 0.001, respectively, Figure 3]. Compared with the subjects in the normal group, those in the mild malnutrition risk group were significantly associated with a higher incidence of adverse events for HI [OR = 2.062, (95% CI = 1.145–3.714), *P* = 0.016; Figure 3]. However, moderate to severe malnutrition risk was not a significant and independent predictor of HI [OR = 2.329 (95% CI = 0.905–5.993), *P* = 0.079; Figure 3]. In univariate analysis for risk of PH, moderate to severe malnutrition risk rather than mild malnutrition risk was identified as a significant factor [OR = 20.750, (95% CI = 2.572–167.381), *P* = 0.004; OR = 2.276, (95% CI = 0.859–6.029), *P* = 0.098, respectively]. The risk for HT was also related to atrial fibrillation, the large size of the infarction, elevated NIHSS score on admission, and anticoagulant therapy before admission.

After initial adjustment for age and gender, patients in the mild malnutrition risk group had a 2.357-fold risk of HT (95% CI = 1.399–3.972, *P* = 0.001), 2.254-fold risk of HI (95% CI = 1.220–4.164, *P* = 0.009) compared to those in the normal group. Meanwhile, it was found that patients with moderate to severe malnutrition risk had an OR (95% CI) of 4.900 (2.136–11.242, *P* < 0.001) for HT, 2.721 (1.003–7.381, *P* = 0.049) for HI after adjusting for age and gender. The difference in the risk of PH was significant between the moderate to severe malnutrition risk group and the normal group when the same analysis was performed [OR = 21.328, (95% CI = 2.605–174.589), *P* = 0.004], while no apparent difference was found between the mild malnutrition risk and the normal group [OR = 2.361, (95% CI = 0.856–6.511), *P* = 0.097]. In multivariate analysis, the association between varying degrees of malnutrition risk and HT remained significant. The OR for HT was higher in patients with a mild malnutrition risk and moderate to severe malnutrition risk [OR = 3.180, (95% CI = 1.139–8.874), *P* = 0.027; OR = 3.960, (95% CI = 1.015–15.453), *P* = 0.048, respectively, Figure 3] after fully adjusted for potential covariates (Model 2: age, gender, history of stroke, hypertension, atrial fibrillation, diabetes, dyslipidemia, smoking status, current alcohol drinking, size of the infarction area, stroke mechanism, SBP, baseline white cell count, fibrinogen, creatinine, baseline NIHSS score, thrombolytic therapy, anticoagulant and antiplatelet use before admission). A similar result was obtained in patients with a mild malnutrition risk group and HI after full adjustments [OR = 3.930, (95% CI = 1.047–14.757), *P* = 0.043]. However, the risk of HI was not significantly higher in the moderate to severe malnutrition risk group [OR = 3.164, (95% CI = 0.415–24.112), *P* = 0.266; Figure 3]. Additionally, the effects of mild malnutrition risk and moderate to severe malnutrition risk on PH were not significant after adjusting for the same potential confounders analyzed above (*P* = 0.740, *P* = 0.505, respectively).

## Discussion

In the present study, we assessed malnutrition risk in patients with AIS at admission by a valid malnutrition score and explored the association between malnutrition risk and HT after ischemic stroke. Our results revealed a significantly increased risk of HT in AIS patients with different malnutrition risks at admission, even after adjustment for a series of potential and known confounders. To the best of our knowledge, few studies have explored and analyzed the association between malnutrition risk and HT in patients with AIS. Notably, our results emphasized the clinical relevance of evaluating the malnutrition risk of patients with AIS at admission and contributed to the advancement of insights into the risk factors of HT.

Several studies have revealed that the prevalence of malnutrition risk in patients with AIS ranged from 1.95 to 20.3%, according to different nutritional assessments (10, 33–35). Additionally, the findings of our research were consistent with these results. The difference in these conclusions may be due to the heterogeneous study population and various evaluation tools of malnutrition risk. Simple and objective risk scoring algorithms have been proposed as practical screening tools to evaluate nutritional status, including the Geriatric Nutritional Risk Index (GNRI) (12) and the CONUT score (11). Previous studies have compared these different assessment tools of malnutrition risk with the European Society for Clinical Nutrition and Metabolism diagnostic criteria for malnutrition (ESPEN-DCM), which is considered the gold standard for malnutrition, and indicated that the two indicators had good accuracy in predicting malnutrition risk (36, 37). Due to different stroke-related complications, such as dysphagia and reduced mobility, it is more challenging to assess nutritional status in patients with stroke. Although GNRI additionally includes anthropometric parameters, the CONUT score encompasses more serum nutritional indicators, which can reflect immune function more objectively and comprehensively based on blood tests (11). Additionally, a single-center prospective study has reported that a substantial proportion of overweight or obese patients measured by BMI were malnourished (38). On account of the objective use of indicators, we used the CONUT score in our study to assess the malnutrition risk.

Previous studies have explored the association between objective malnutrition scores and the occurrence of adverse outcomes. A higher CONUT score could predict cardiovascular death and all-cause mortality in the general population, according to a prospective study in the United States (39). López Espuela et al. have demonstrated a significant association between moderate to severe malnutrition risk and increased all-cause death at 3 months in Spanish patients with AIS (40). Also, a study in Japan found that patients with moderate to severe malnutrition risk were independently related to poor 3-month functional outcomes (41). In two other recent studies, nutritional status assessed by the CONUT score was independently related to the poor functional outcome in ischemic stroke patients with large vessel occlusion after endovascular thrombectomy (42) and associated with a higher risk of mortality at 3 months in patients with ischemic stroke undergoing intravenous thrombolysis (43). As two main methods of reperfusion treatments for acute ischemic stroke (44), intravenous thrombolysis was considered a risk factor for HT in patients with AIS (45, 46), while the effect of endovascular thrombectomy on HT in ischemic stroke still needs further confirmation (47, 48). A recent study suggested a significant association between moderate to severe malnutrition risk assessed by objective nutritional tools and poor long-term prognosis in large-scale Chinese patients with AIS (10). However, few studies have yet to explore the malnutrition risk and its relationship with hemorrhagic transformation in patients with AIS. Our study further indicated that malnutrition risk was significantly associated with HT even after adjusting for potential risk factors. In this study, patients with mild and moderate to severe malnutrition risk had higher risks of HT compared to normal patients [OR = 3.180, (95% CI = 1.139–8.874), *P* = 0.027; OR = 3.960, (95% CI = 1.015–15.453), *P* = 0.048, respectively]. However, there was no significant association between moderate to severe malnutrition risk and increased risk of HI or PH based on the adjusted multivariate regression analysis. Interestingly, we found patients with mild malnutrition risk were more likely to undergo HI [OR = 3.930, (95% CI = 1.047–14.757), *P* = 0.043]. We supposed that higher malnutrition risk patients could be more likely to experience PH, representing a more severe radiological category and poor clinical outcomes in patients with stroke. However, the association between varying degrees of malnutrition risk and PH was insignificant after full adjustments. Due to the limited number of patients in our study, we hope more studies can provide further evidence to confirm these results.

Several factors constituting the nutritional status score may account for the observed association between malnutrition index and HT. First, serum albumin functions as a major plasma antioxidant defense and constitutes the primary oxygen radical trapping (49–51). As a specific inhibitor of endothelial cell apoptosis, serum albumin can protect against exogenous and endogenous oxidation (15, 52). Reactive oxygen species seem to perform an important role in early HT, which might lead to petechial hemorrhage because of damage to the neurovascular unit at the capillary level (19). A previous study has demonstrated that low serum albumin is an independent predictor of HT in ischemic stroke patients with intravenous thrombolysis (53). Second, relative reductions in lymphocytes reflect activation of the sympathetic excitation and stress response, which can aggravate ischemic injury through the increased production of proinflammatory cytokines (54). A large number of studies in recent years have confirmed the predictive value of the neutrophil-to-lymphocyte ratio for poor outcomes in patients with ischemic stroke, including the risk of hemorrhagic transformation (55, 56). The effect of total cholesterol on the prognosis of stroke was still controversial. A higher total cholesterol level was reported to be associated with poor outcomes after stroke and a lower total cholesterol level (57–59). In this study, the total cholesterol level did not show statistical significance in univariate analysis, which may be linked to varying degrees of statin treatment for stroke.

There are several limitations to our study. First, this study was a single-centered and retrospective analysis, which could not prove cause and effect. Second, due to the unavailability of necessary variables, we did not make a comparison between different nutritional scores and were unable to assess malnutrition status by gold standards, including the European Society for Clinical Nutrition and Metabolism diagnostic criteria for malnutrition. However, the CONUT score used in this study was reported to be relatively accurate in evaluating the malnutrition risk and was of great importance for the guidelines of clinical therapy. We hope more studies can further investigate the variation among different evaluation tools and verify the findings of our study. Third, we take the infarct size as a categorical variable in our analysis owing to the limitations of the experimental condition. It is more precise to calculate the infarct size using the Alberta Stroke Program Early CT Score (ASPECTS) system by trained radiologists. Additionally, the sample size of this study was limited. More studies need to be conducted to further confirm the relationship between malnutrition risk and HT. Finally, we evaluated malnutrition risk only at admission but not at dynamic observation. It is unclear whether malnutrition status changes over time could influence the risk of hemorrhagic transformation. Further study is warranted to fully understand the influence of nutrition interventions on malnourished patients.

## Conclusion

In conclusion, this study demonstrated that malnutrition risk, measured by the CONUT score, was significantly associated with an increased risk of hemorrhagic transformation in patients with AIS.

## Data availability statement

The datasets presented in this article are not readily available because the research data is confidential. Requests to access the datasets should be directed to the corresponding author.

## Ethics statement

The studies involving human participants were reviewed and approved by the Institutional Review Board and Research Ethics Committee of the Second Affiliated Hospital and Yuying Children's Hospital of Wenzhou Medical University. Written informed consent was not provided because this study was retrospective and all included data were anonymous.

## Author contributions

C-XY and B-LH designed the study. C-XY and X-YC interpreted the data. C-XY wrote the manuscript. Y-NZ prepared the figures. Y-NZ and X-YC performed the statistical analyses. B-LH supervised the study. All authors have made an intellectual contribution to the manuscript and approved the submission.

## Funding

This study was supported by grants from the projects of the National Natural Science Foundation of China (81873799, 81771267) and the Clinical Scientific Research Foundation of the Second Affiliated Hospital of Wenzhou Medical University (SAHoWMU-CR2017-01-212).

## Conflict of interest

The authors declare that the research was conducted in the absence of any commercial or financial relationships that could be construed as a potential conflict of interest.

## Publisher's note

All claims expressed in this article are solely those of the authors and do not necessarily represent those of their affiliated organizations, or those of the publisher, the editors and the reviewers. Any product that may be evaluated in this article, or claim that may be made by its manufacturer, is not guaranteed or endorsed by the publisher.
